# Cost-effectiveness analysis of atezolizumab in advanced triple-negative breast cancer

**DOI:** 10.1186/s12913-020-05445-6

**Published:** 2020-06-24

**Authors:** Lee Cheng Phua, Soo Chin Lee, Kwong Ng, Mohamed Ismail Abdul Aziz

**Affiliations:** 1 Agency for Care Effectiveness, Ministry of Health, Singapore, 14 College Road, Singapore, 169853 Singapore; 2 Department of Haematology-Oncology, National University Cancer Institute, Singapore, Singapore

**Keywords:** Atezolizumab, Immunotherapy, Programmed death ligand-1, Triple-negative breast cancer, Cost-effectiveness, Partitioned-survival

## Abstract

**Background:**

The IMpassion130 trial demonstrated that adding atezolizumab to nanoparticle albumin-bound (nab)-paclitaxel improved the survival of patients with untreated, advanced, programmed death ligand 1 (PDL1)-positive triple-negative breast cancer (TNBC). In view of the high cost of immunotherapy, it is important to examine its value with respect to both benefits and costs. In this study, the cost-effectiveness of atezolizumab/nab-paclitaxel combination therapy relative to nab-paclitaxel monotherapy was evaluated for the first-line treatment of advanced, PDL1-positive TNBC, from a healthcare system perspective.

**Methods:**

A three-state partitioned-survival model was developed to compare the clinical and economic outcomes of treatment with atezolizumab/nab-paclitaxel combination therapy with nab-paclitaxel monotherapy in patients with advanced TNBC. Clinical data were obtained from the IMpassion130 trial and extrapolated to 5 years. Health state utilities were retrieved from the literature, while direct costs (in Singapore dollars, S$) were sourced from public healthcare institutions in Singapore. The primary outcomes of the model were life years (LYs), quality-adjusted LYs (QALYs), costs and incremental cost-effectiveness ratios (ICERs). One-way and probabilistic sensitivity analyses and scenario analyses were conducted to explore the impact of specific assumptions and uncertainties.

**Results:**

Adding atezolizumab to nab-paclitaxel resulted in an additional 0.361 QALYs (0.636 LYs) at an ICER of S$324,550 per QALY gained. The ICER remained high at S$67,092 per QALY even when atezolizumab was priced zero. One-way sensitivity analysis showed that the ICER was most sensitive to variations in the cost of atezolizumab and the time horizon. Scenario analyses confirmed that the ICERs remained high even under extremely favourable assumptions.

**Conclusions:**

Given the exceedingly high ICER, adding atezolizumab to nab-paclitaxel was unlikely to represent good value for money for the treatment of advanced PDL1-positive TNBC. Our findings will be useful in informing funding policy decisions alongside other considerations such as comparative effectiveness, unmet need and budget impact.

## Background

Breast cancer is the most common malignancy and the leading cause of cancer-related death among women worldwide, with over 2 million new cases and 626,679 deaths reported in 2018 [1]. Triple-negative breast cancer (TNBC) is a subtype of breast cancer characterized by minimal expression of estrogen and progesterone receptor and an absence of human epidermal growth factor receptor-2 (HER2) overexpression. It constitutes approximately 15–20% of all breast carcinomas, amounting to more than 300,000 new cases per year worldwide, and has been associated with an aggressive clinical course and poor overall survival of not more than 18 months [2, 3]. Unlike tumours overexpressing hormone receptors or HER2, TNBC is not amenable to treatment with endocrine therapy or anti-HER2 targeted therapy [4]. As such, systemic therapeutic options have historically been limited to cytotoxic chemotherapy such as taxanes or anthracyclines. Given the paucity of treatment options and poor prognosis of the disease, the development of more effective treatment strategies for TNBC has been an area of active research.

In recent years, a growing body of evidence has pointed towards the immunogenic nature of TNBC and the potential role of immune modulation as a therapeutic approach for the cancer. Patients with TNBC have shown a higher incidence of possessing a robust tumour T-cell infiltrate than those with other breast cancer subtypes [5]. Immune checkpoint proteins such as programmed death ligand-1 (PDL1) have also been found to be significantly upregulated in TNBC. As such, clinical efforts have been directed towards evaluating the use of immune checkpoint inhibitors to potentiate the host’s immune response against the cancer and in turn improve the outcomes of patients with TNBC.

IMpassion130 was one of the first phase III trials of immune checkpoint inhibitors conducted in patients with previously untreated, metastatic or unresectable locally advanced TNBC [6]. The multicentre, double-blind, randomized controlled trial conducted in 41 countries showed that the combination of atezolizumab, a PDL1 inhibitor, and nanoparticle albumin-bound (nab)-paclitaxel significantly improved progression-free survival (PFS) by 20% compared with nab-paclitaxel alone in the intention-to-treat population (median PFS 7.2 versus 5.5 months) and by 38% in the pre-defined subgroup of patients with PDL1 expression on ≥1% of tumour-infiltrating immune cells (median PFS 7.5 versus 5.0 months). While the combination was not shown to significantly improve overall survival (OS) in the intention-to-treat population, it demonstrated an unprecedented OS benefit of 9.5 months in the PDL1-positive subgroup population (median OS 25.0 versus 15.5 months). The promising results of the study have led the US Food and Drug Administration to grant accelerated approval for using atezolizumab in combination with nab-paclitaxel in adults with metastatic or unresectable locally advanced, PDL1-positive TNBC [7]. The National Comprehensive Cancer Network (NCCN) clinical practice guideline has also recommended the combination therapy as the preferred regimen for treating PDL1-positive, recurrent or stage IV TNBC [8].

Despite the potential clinical benefits, much concerns arose regarding the significant increased costs associated with cancer immunotherapies such as atezolizumab. To our knowledge, there has been no published study that assessed the value of atezolizumab with respect to its costs and effectiveness for the treatment of TNBC. Therefore, the objective of the present study was to evaluate the cost effectiveness of atezolizumab/nab-paclitaxel combination therapy relative to nab-paclitaxel monotherapy for first-line treatment of metastatic or unresectable locally advanced, PDL1-positive TNBC, from a healthcare system perspective. The results of this study will provide timely information to health policy makers regarding the economic value of atezolizumab-based regimen and support funding decisions alongside other relevant considerations.

## Methods

### Overview of model

**Fig. 1:**
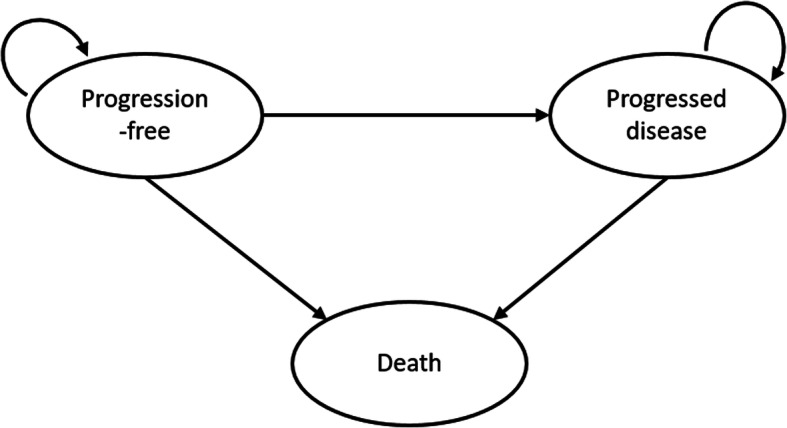
Schematic of the partitioned survival model with three health states, namely, progression-free, progressed disease and death. Arrows represent possible transitions through the health states

A partitioned survival model was developed in Microsoft Excel to evaluate the cost-effectiveness of atezolizumab plus nab-paclitaxel compared with nab-paclitaxel alone for first-line treatment of metastatic or unresectable locally advanced, PDL1-positive TNBC. In a partitioned survival model, the proportion of patients in each health state at each time point was derived from the OS and PFS curves to inform the benefits accrued and costs incurred over the time horizon of the model. Three health states were considered, namely progression-free (PF), progressed disease (PD) and death (Fig. 1).

The model followed patients weekly (with half cycle corrections) over a time horizon of 5 years. The time horizon was considered sufficient to capture the costs and outcomes over the lifespan of the majority of patients, based on a reported 5-year survival of less than 10% observed in women with Stage IV triple negative breast cancer from the US Surveillance, Epidemiology and End Results (SEER) population-based cancer registry [9].

### Patients and intervention

Patients with previously untreated metastatic or unresectable locally advanced, histologically documented TNBC who have PDL1 expression on ≥1% of tumour-infiltrating immune cells (expressed as a percentage of tumour area) were assessed in this model. The pivotal phase III IMpassion130 trial assessed both the intention-to-treat and a priori planned PD-L1 positive subgroup of patients for a median follow-up of 12.9 months [6]. The latter was being modelled in this analysis consistent with FDA-registered indication. The treatment dosing regimen was in line with the trial. Atezolizumab was administered intravenously at a fixed dose of 840 mg, on days 1 and 15 while nab-paclitaxel was given at a dose of 100 mg/m^2^ on days 1, 8, and 15 of every 28-day cycle. Nab-paclitaxel was combined with atezolizumab to potentially enhance the response to immune checkpoint inhibition. It was preferred over solvent-based paclitaxel because the glucocorticoid premedication administered alongside solvent-based paclitaxel had been hypothesized to reduce immunotherapy activity. All patients continued their respective regimens until progression or an unacceptable level of toxic effects occurred. After progression, a proportion of patients were treated with subsequent-lines regimen until palliative care or death. There was no cross-over option for patients in either treatment arm, i.e. there was no option for patients receiving placebo to cross-over to receive atezolizumab.

### Outcomes

Cost-effectiveness analyses were conducted from the perspective of the Singapore healthcare system, which comprises a combination of payers including the government, insurance providers and patients [10, 11]. The government in Singapore provides subsidies and financial assistance to eligible patients treated at public healthcare institutions, for drugs listed on the Standard Drug List and the Medication Assistance Fund [12]. The outcomes-of-interest were costs, life years (LYs), quality-adjusted life years (QALYs) and incremental cost-effectiveness ratio (ICER). Costs and QALYs were discounted at 3% per annum [10].

### Model parameters

#### Clinical efficacy data

Clinical efficacy data were derived from the pivotal phase III IMpassion130 trial (first interim analysis) [6]. The published Kaplan Meier (KM) OS and PFS curves were digitized using a validated graphical digitizer (WebPlotDigitizer version 4.2; Ankit Rohatgi, CA, USA) [13]. Then, individual patient time-to-event data were reconstructed from the digitized KM curves using an algorithm developed by Guyot et al. [14] Parametric survival curve fitting was subsequently performed on the data to model the outcomes beyond the trial duration. The fitting was performed separately on data from the two treatment arms to avoid proportional hazards assumption. Candidate functions for the parametric extrapolation were Weibull, exponential, Gompertz, log-normal, log-logistic and generalized gamma distributions. The Weibull and exponential distributions were found to best fit the OS and PFS data, respectively, based on the assessments of clinical plausibility by local oncologist and goodness-of-fit given by the Akaike Information Criterion (AIC) value.

Internal validation of the model was conducted by comparing the modelled clinical outcomes with empirical trial data in terms of median OS and PFS and 2-year OS. Additionally, as the updated OS results from the second interim analysis of the trial were published at the time of completion of the model [15, 16], these results were also used for internal validation of the model.

At each cycle of the model, data were processed to ensure that (1) the modelled death rate was higher than or at least equal to the general population mortality, based on Singapore life table [17], and (2) the proportion of patients who had disease progression or died, given by the PFS curve, was smaller than or at least equal to the proportion who died, given by the OS curve.

#### Utilities

Health state utilities are numerical values that represent an individual’s preferences for different health-related outcomes, ranging from 0 (representing a state of death) to 1 (representing a state of perfect health). These values are multiplied by the time spent in the health state (i.e. number of LYs) to produce the outcome of QALYs in the model.

Utilities of 0.715 and 0.443 were used for the PF and PD states, respectively, based on a primary utility study by Lloyd et al. [18] The choice of this study was consistent with prior economic analyses [19–22]. Disutilities due to adverse events (AEs) were not included in the model as the reported differences in incidences of AEs between the two treatment groups were not considered significant enough to result in differences in quality of life.

#### Costs

Direct healthcare costs including costs of drugs, intravenous drug administration, doctor consultation visits, blood tests, scans and palliative care were considered in the analysis. The average costs to patients at public healthcare institutions in Singapore were used. (Table 1). The doses of first-line drugs (atezolizumab and nab-paclitaxel) were in line with the IMpassion130 clinical trial, while that of subsequent-lines drugs were informed by licensed dosing regimens or the NCCN clinical guideline. An average body surface area (BSA) of 1.6m^2^, estimated by local oncologist, was assumed in drug dosing calculations. The management of drug-related AEs was not expected to result in significant differences in resource consumption between the two treatment arms, based on clinical opinion. Therefore, AE-related costs were not included in the model. All costs were presented in Singapore dollars (S$).

**Table 1:** Cost inputs

Component	Cost (S$)	Reference
**First-line drugs, per vial**		
Atezolizumab (840 mg-vial)	4171.56	[f],[g]
Nab-paclitaxel (100 mg-vial)	667.81	[f]
**Subsequent-lines drug regimens, per week**		
Cost of subsequent-lines regimens after 1st-line treatment with atezolizumab plus nab-paclitaxel	69.31	[f],[h]
Cost of subsequent-lines regimens after 1st-line treatment with nab-paclitaxel	90.59	[f],[h]
**Intravenous drug administration, per administration**^a^		
Drug preparation fee by pharmacy	52.80	[f]
Facility fee/chair time	272.20	[f]
**Disease monitoring and management, per session**		
Doctor’s clinic consultation ^b^	74.57	[f]
Computed tomography (CT) scan ^c^	940.00	[f]
Liver function test ^b^	71.30	[f]
Renal panel ^b^	62.80	
Full blood count ^d^	26.46	[f]
Thyroid function test (for patients receiving atezolizumab) ^b^	226.28	[f]
**Palliative care, per month**		
Cost of palliative care ^e^	3210.90	[i]

^a^The frequencies of administration of first-line drugs (atezolizumab and nab-paclitaxel) were in line with the IMpassion130 clinical trial, while that of subsequent-lines drugs were informed by licensed dosing regimens or NCCN clinical guideline

^b^Performed every 4 weeks for patients receiving anti-cancer therapies, according to local practice

^c^CT scan was performed every 10 weeks for patients receiving anti-cancer therapies (CT scans are typically done every 8–12 weeks in local practice)

^d^Test was performed weekly during treatment with nab-paclitaxel. For patients on other therapies, the test was performed every 4 weeks, according to local practice

^e^It was assumed that palliative care was provided for 1 month before death

^f^The costs of drugs, drug administration or disease management were estimated from the average costs to patients at public healthcare institutions in Singapore (2018)

^g^The cost of atezolizumab 840 mg-vial was extrapolated from that of the 1200 mg-vial, assuming linear pricing

^h^Subsequent-lines drugs provided to > 5% of patients in at least one of the two arms within the IMpassion130 trial were considered. Drug costs were calculated based on the dosing regimens recommended by package inserts or NCCN clinical guideline and an average BSA of 1.6m^2^. The average cost was weighted according to the proportion of use of each drug reported in the trial

^i^The average cost of palliative care (weighted according to the proportion of patients receiving inpatient hospice care or home-based care) was obtained from one hospice centre in Singapore

### Sensitivity and scenario analyses

One-way sensitivity analyses were performed to explore the impact of uncertain model parameters on the ICER. The health state utilities and cost of atezolizumab were varied arbitrarily by ±20%. Discount rates were varied from 0 to 5% for both costs and outcomes while the time horizon was varied from 3 to 10 years.

Probabilistic sensitivity analysis was performed using 15,000 Monte Carlo simulations to further assess the robustness of the results. Uncertainty in each parameter was represented by an appropriate probability distribution that corresponded with the nature of the variable. Utility values were assumed to follow the beta-distribution, while parameter values of survival functions for OS and PFS were sampled from the multivariate normal distribution using the Cholesky decomposition matrix of the Weibull and exponential distributions, respectively. Unlike countries such as the United Kingdom where cost-effectiveness thresholds are used in making resource allocation decisions [23], there is no explicit fixed cost-effectiveness threshold in Singapore, as the decision to subsidise a health technology is informed by multiple factors besides cost-effectiveness, such as comparative effectiveness, unmet need and budget impact. As such, a cost-effectiveness acceptability curve was generated to present the probability of each intervention being cost-effective across a range of cost-effectiveness thresholds.

Scenario analyses were conducted to probe the effects of uncertainty in the assumptions about treatment costs and utilities. Extreme scenarios with highly favourable assumptions (utilities of 1 and substantial price reduction of atezolizumab and nab-paclitaxel) were tested to assess the lower limit of the ICER. Costs based on local treatment options (for instance, alternative use of subsequent-lines treatments and limited use of nab-paclitaxel for 6 months) were also evaluated. To explore the possibility of cure, the presence or absence of a cure fraction (a proportion of patients who were cured of disease) in the arm treated with atezolizumab plus nab-paclitaxel was tested by fitting a mixture-cure model described by Lambert et al. [24]

## Results

### Survival regression

**Fig. 2:**
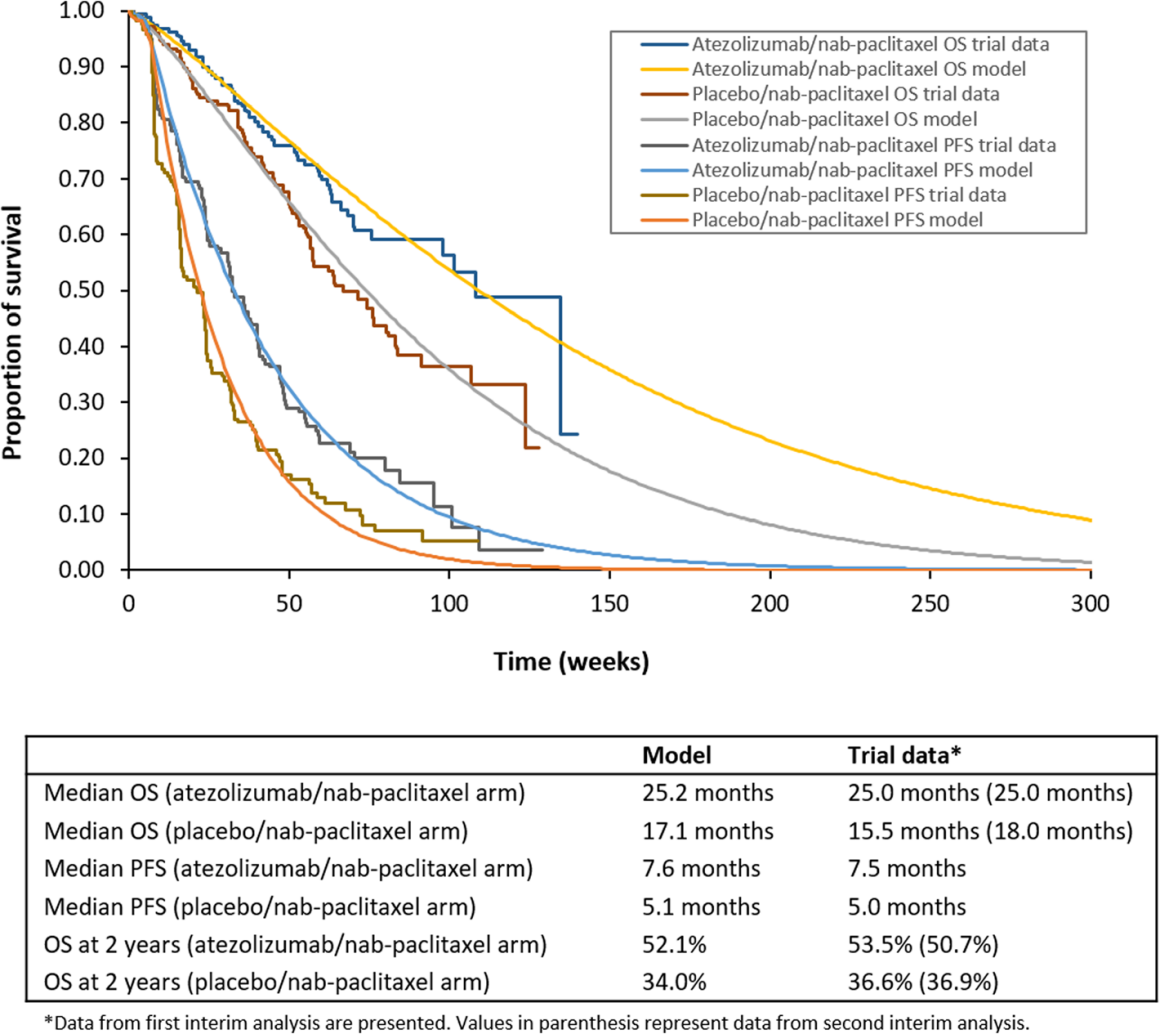
Internal validation of model. Kaplan Meier OS and PFS curves from IMpassion130 trial and model-generated OS and PFS curves are shown. Modelled clinical outcomes showed agreement with empirical data from the IMpassion130 trial in terms of median OS and PFS and 2-year OS

Modelled clinical outcomes were in line with empirical trial data in terms of median OS and PFS and 2-year OS, confirming the internal validity of the parametric regression model (Fig. 2).

### Base case cost-effectiveness

Compared with nab-paclitaxel monotherapy, the combination of atezolizumab and nab-paclitaxel led to a mean gain of 0.636 LYs and 0.361 QALYs per person, over a time horizon of 5 years (Table 2). These benefits were achieved at an incremental cost of S$117,060. As a result, the addition of atezolizumab to nab-paclitaxel was associated with a deterministic base-case ICER of S$183,965 per LY gained and S$324,550 per QALY gained.

**Table 2:** Summary of costs and benefits of atezolizumab plus nab-paclitaxel versus nab-paclitaxel (base-case)

	Atezolizumab/nab-paclitaxel	Nab-paclitaxel	Incremental
PFLYs	0.856	0.566	0.290
LYs	2.308	1.672	0.636
QALYs	1.255	0.895	0.361
Total Costs (S$)	173,623	56,563	117,060
Drug and drug administration costs, first-line	148,311	36,688	111,623
Drug and drug administration costs, subsequent-lines	8551	8230	321
Disease monitoring and management costs	14,107	8645	5461
Palliative care costs	2654	3000	− 345
ICER (S$ per LY gained)			183,965
ICER (S$ per QALY gained)			324,550

*PFLY* progression-free life year, *LY* life year, *QALY* quality-adjusted life year, *ICER* incremental cost-effectiveness ratio

### Sensitivity and scenario analysis

**Fig. 3:**
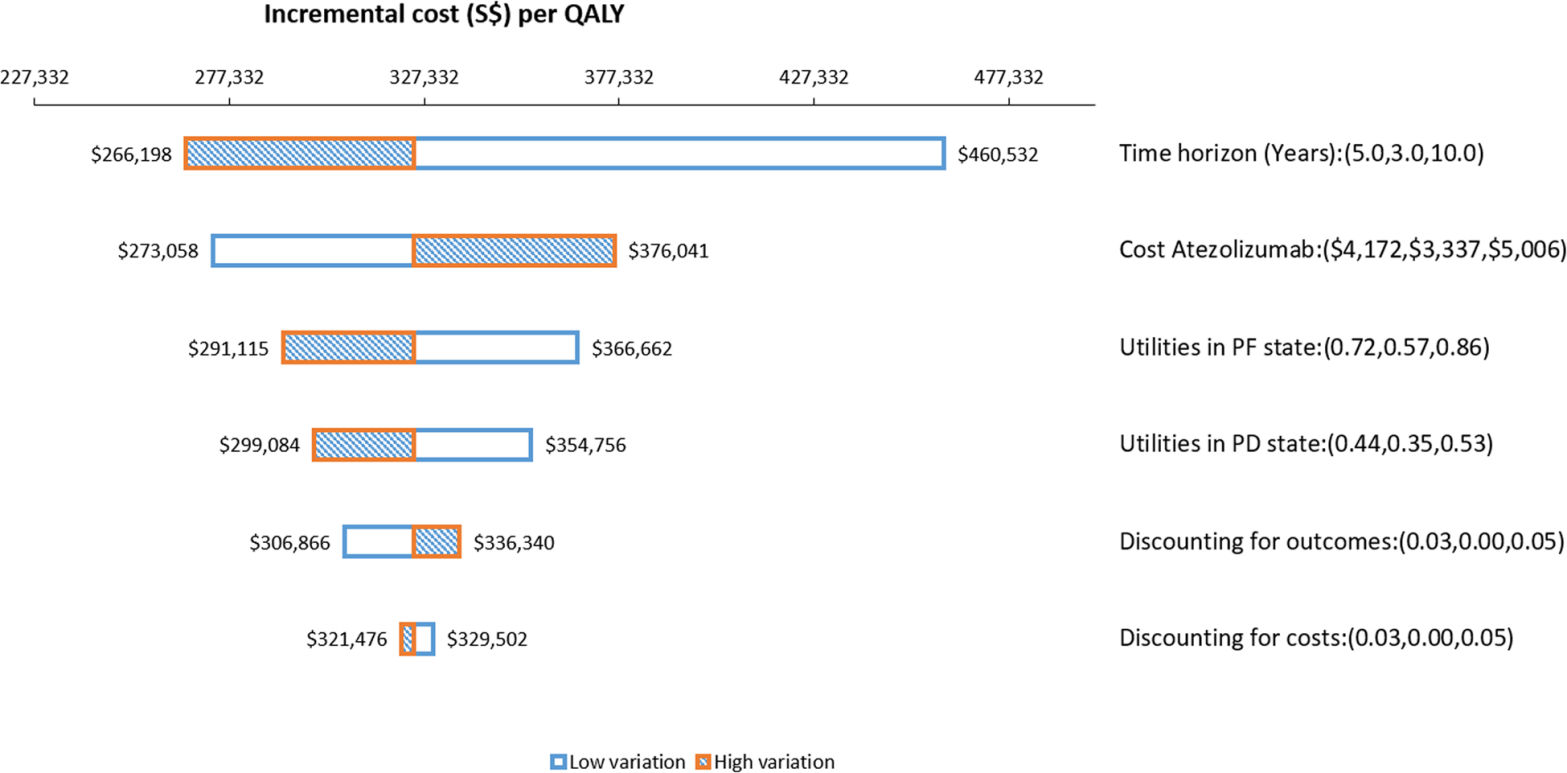
Tornado diagram representing results of one-way sensitivity analysis. Model parameters were varied within the range of values shown in parenthesis. QALY: quality-adjusted life year; PF: progression-free; PD: progressed disease

The ICER was most sensitive to the time horizon of the model, as shown by results of deterministic one-way sensitivity analyses (Fig. 3). Shortening the time horizon to 3 years raised the ICER considerably to S$460,532 per QALY gained while extending it to 10 years lowered the ICER to S$266,198 per QALY gained. The ICER was also sensitive to variations in the cost of atezolizumab; when the cost of atezolizumab was varied by ±20%, the ICER ranged widely from S$273,058 to S$376,041 per QALY gained.

To explore the potential presence of a cure fraction among patients treated with atezolizumab immunotherapy, a mixture-cure model was used to fit the survival data of the patients in the atezolizumab/nab-paclitaxel treatment arm. Our results did not show any clear evidence of the plausibility of a cure fraction in relation to OS or PFS among this group of patients, as the point estimate and confidence interval of the statistically-derived cure proportion included negative values [cure proportion: − 1.4% (95% CI -117.6 – 114.8%, *p* = 0.981) and − 5.4% (95% CI -23.6 – 12.8%, *p* = 0.562), respectively]. This observation supported the use of a standard parametric regression approach to model the survival data of these patients.

Scenario analyses confirmed that the ICER remained exceedingly high even under extremely optimistic assumptions (Table 3). When perfect health state utilities were assigned to both PF and PD states, the ICER was S$183,965 per QALY gained. When the cost of atezolizumab was reduced by 50, 70 and 90%, the ICERs were S$195,821, S$144,329 and S$92,838 per QALY gained, respectively. When the cost of both first-line therapies, atezolizumab and nab-paclitaxel, were reduced by 90%, the ICER was S$55,185. Interestingly, even when cost of atezolizumab was dropped to zero price, the ICER remained high at S$67,092 per QALY. The ICERs were also consistently high when different cost inputs were tested to reflect alternative treatment options in local practice.

**Table 3:** Results of scenario analyses

Assumptions	Incremental QALY	Incremental cost (S$)	ICER (S$/QALY)
**Utilities**			
Progression-free utility 1.0 ^a^	0.443	117,060	264,097
Progressed disease utility 1.0 ^a^	0.554	117,060	211,392
Both progression-free and progressed disease utilities 1.0 ^a^	0.636	117,060	183,965
**Cost**			
Subsequent lines regimens according to local clinical practice ^b^	0.361	124,154	344,216
Nab-paclitaxel stopped at 6 months in the atezolizumab/nab-paclitaxel group ^c^	0.361	96,018	266,210
Atezolizumab at 50% cost	0.361	70,630	195,821
Atezolizumab at 30% cost	0.361	52,057	144,329
Atezolizumab at 10% cost	0.361	33,485	92,838
Atezolizumab alone free ^a^	0.361	24,199	67,092
Nab-paclitaxel alone free^a^	0.361	101,971	282,714
Both atezolizumab and nab-paclitaxel at 50% cost	0.361	63,085	174,903
Both atezolizumab and nab-paclitaxel at 30% cost	0.361	41,495	115,044
Both atezolizumab and nab-paclitaxel at 10% cost	0.361	19,905	55,185
Both atezolizumab and nab-paclitaxel free ^a^	0.361	9109	25,256

^a^Highly favourable scenarios to assess the lower limit of ICER

^b^The average cost of subsequent-lines treatments was calculated based on the cost of drugs commonly used in local practice (capecitabine, eribulin and doxorubicin), weighted according to the proportion of use

^c^Cost of drugs and the associated administration and monitoring fees were calculated assuming nab-paclitaxel was given for a maximum of 6 months (in line with potential clinical practice)

**Fig. 4:**
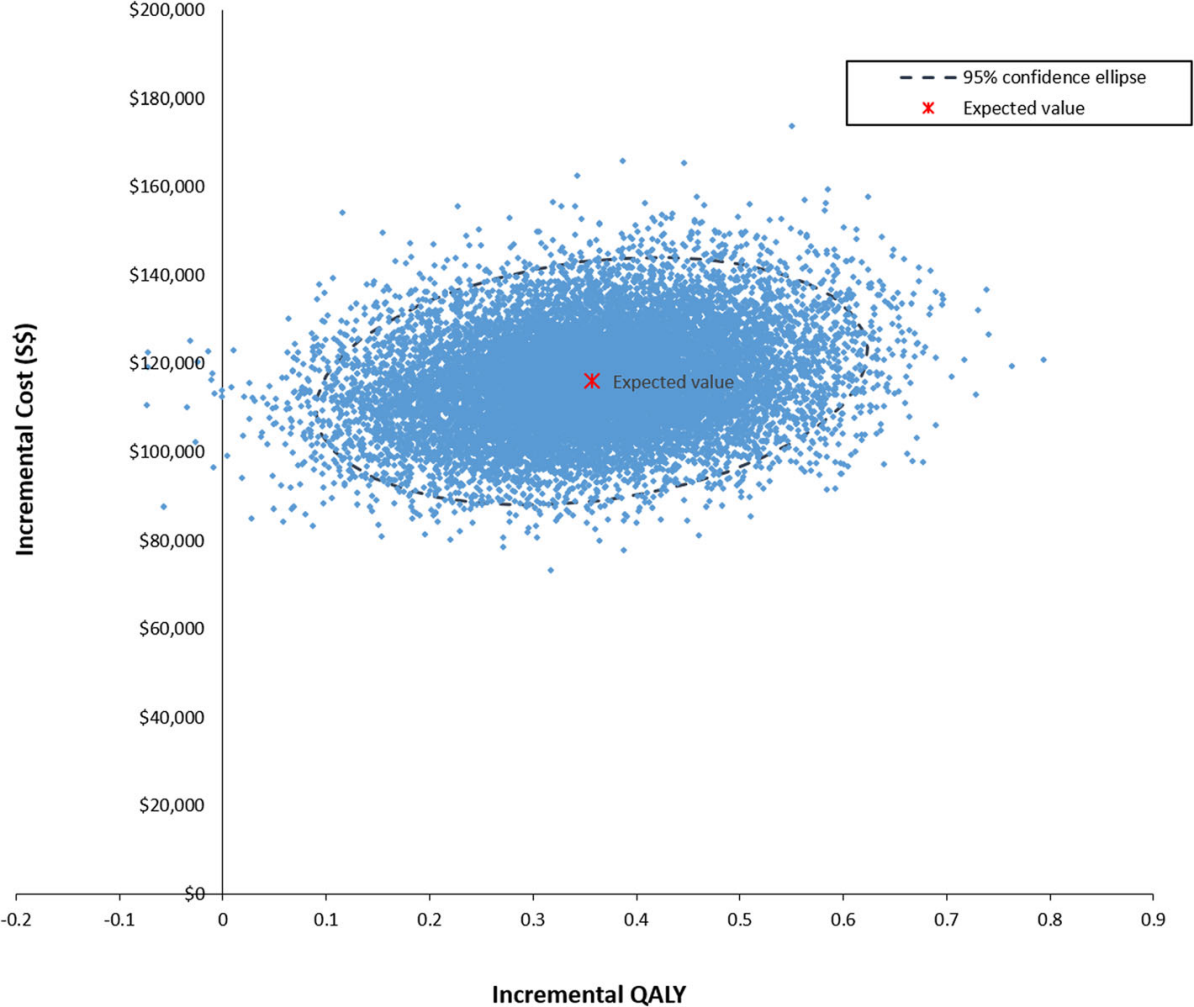
Incremental cost-effectiveness scatterplot of 15,000 Monte Carlo simulations. Each dot represents the ICER for 1 simulation

**Fig. 5:**
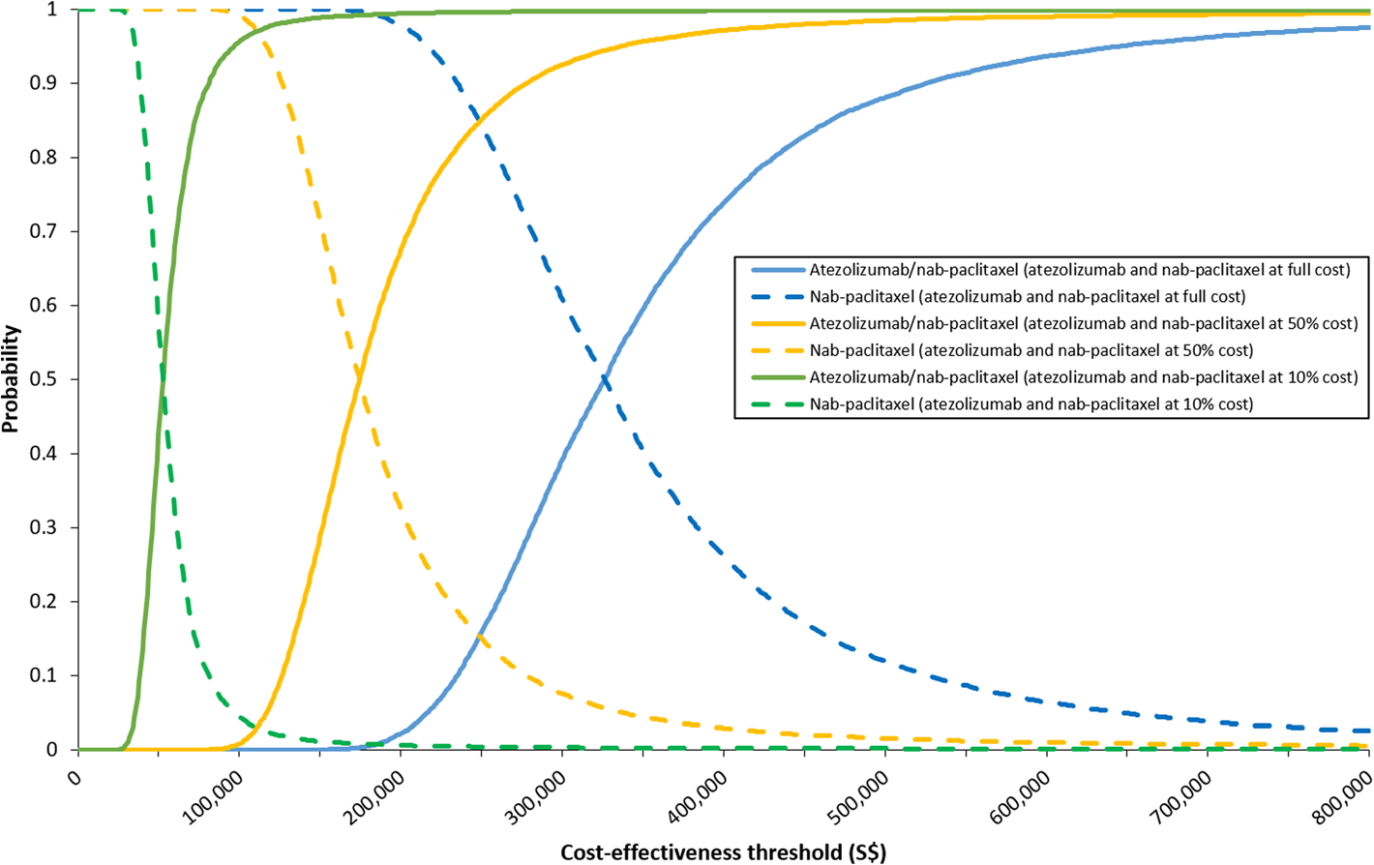
Cost-effectiveness acceptability curves showing the probability of each intervention being cost-effective over a range of cost-effectiveness thresholds. Separate curves were presented for different cost scenarios

Probabilistic sensitivity analysis showed a mean ICER of S$325,416 per QALY, similar to the deterministic base-case result (Fig. 4). The combination of atezolizumab and nab-paclitaxel had less than 1% likelihood of being cost-effective between a threshold range of S$0 and S$188,333 per QALY (Fig. 5). The combination therapy was more likely to be a cost-effective treatment option compared with nab-paclitaxel monotherapy only when the threshold rose above S$326,353 per QALY. This value was decreased to S$174,409 and S$52,620 upon cost reductions of both first-line drugs by 50 and 90%, respectively.

## Discussion

The regulatory approval of the use of atezolizumab immunotherapy for the treatment of TNBC represented a landmark therapeutic development given the limited treatment options available for this highly aggressive subtype of breast cancer. While the availability of the drug is seen to fulfil an unmet clinical need, it is of considerable interest to policy makers whether the health benefits conferred by the drug justifies the additional costs. The present study is the first to examine the cost-effectiveness of the first-line use of atezolizumab/nab-paclitaxel relative to nab-paclitaxel in patients with advanced, PDL1-positive TNBC.

Results from our study suggested that, from a healthcare system perspective, treatment with atezolizumab/nab-paclitaxel did not represent good value for money. The findings were robust in probabilistic simulations, and across a range of plausible input values. The unfavourable ICER was driven largely by the high cost of atezolizumab. Particularly, the total cost per person incurred from the administration of atezolizumab/nab-paclitaxel was substantially higher than that of nab-paclitaxel (S$148,311 over 10.3 months versus S$36,688 over 6.8 months). Weighed against the marginal gain in QALYs (0.361), the addition of atezolizumab was unlikely to be cost-effective given the high cost of the drug.

Scenario analyses highlighted that even when extreme assumptions of near-perfect health state utilities or appreciable price reduction of atezolizumab were applied, the addition of atezolizumab did not provide reasonable value for the money spent. Interestingly, certain scenarios revealed that the ICER was affected markedly not only by the cost of atezolizumab but also that of nab-paclitaxel. It was found that even if atezolizumab was priced at zero cost, the ICER remained prohibitive at S$67,092 per QALY gained. A drastic reduction in the cost of both drugs was required before cost-effectiveness may potentially be achieved. The cost-effectiveness of combination drug regimens for oncology treatment has been a topic of recent discussion. The UK National Institute for Health and Care Excellence (NICE)- commissioned report by the Decision Support Unit (DSU) examined various scenarios in which clinically effective new technologies were not cost effective at zero price [25]. One of the reasons for such an occurrence was in the context of combination regimens in which the existing treatment (backbone to which new technology is co-administered) was itself not cost-effective. As highlighted in the DSU report, a possible explanation was that in a non-curative setting where treatment was continued until disease progression, prolonging PFS implied lengthening the time spent accruing costs for expensive drugs. This is exacerbated if the cost of the backbone treatment, in this case nab-paclitaxel, is high to begin with. This apparent paradox accounted for the low likelihood that the addition of atezolizumab could be cost-effective in most scenarios.

Our findings of unfavourable cost-effectiveness of atezolizumab resonated with prior analyses in other types of cancers. In the first-line treatment of extensive-stage small-cell lung cancer (SCLC), the addition of atezolizumab to carboplatin and etoposide led to a high ICER of US$528,810 per QALY gained in the United States [26]. Similarly, in the first-line treatment of metastatic, non-squamous non-small cell lung cancer (NSCLC), the addition of atezolizumab to the combination of bevacizumab, carboplatin and paclitaxel was associated with an ICER as high as US$568,967 per QALY gained [27]. Even when the use of atezolizumab was restricted to patients with high PDL1 expression (on 50% or more of tumour cells or 10% or more of tumour-infiltrating immune cells), the ICER remained high at US$464,703 gained per QALY gained. The results were similar, albeit slightly improved, when atezolizumab was used for the second-line treatment of advanced NSCLC in patients who progressed after platinum-doublet chemotherapy. The drug resulted in an ICER of C$142,074 per QALY gained compared with docetaxel in Canada and US$215,802 per QALY gained in the United States [28, 29]. In the latter case, limiting the use of atezolizumab to a small subset of patients with high PDL1 expression was required before the ICER could be reduced to US$76,459 per QALY gained. Overall, the opportunity cost of adopting atezolizumab-based therapy in the various cancer settings investigated to date far exceeded the benefits to patients.

Several limitations should be considered when interpreting the results of our study. First, as with most cost-effectiveness analyses of new oncology treatments, long-term survival data were not available in the pivotal trial at the point of the economic evaluation. To project the costs and relevant health outcomes over a 5-year time horizon, necessary assumptions were made regarding the survival distribution beyond the trial duration. The uncertainty in the parametric assumptions were partially addressed by validating the modelled outcomes against currently available empirical data but further validation will be required as longer-term data emerge from the IMpassion130 trial or observational studies. Nonetheless, the results of the current study offer important and timely information to support policy makers in their initial funding decisions.

Second, in the absence of utility data collected directly in the IMpassion130 trial, estimates from a primary utility study were used. Of note, the utility study by Lloyd et al had used vignettes to describe the health states, an approach that had often been criticized for not being able to adequately characterize the varied distribution of symptoms, physical functioning, pain and feelings of well-being among patients [30]. Furthermore, the vignettes were developed from a literature review and interviews with clinicians without direct input from patients. Accuracy of the description of health states may therefore be compromised. In addition, the study was designed to elicit the utilities of the overall metastatic breast cancer population instead of patients with triple-negative subtype. Given the disparate disease profile, the health-related quality of life of the two groups of patients may possibly differ. As a result of these factors, large uncertainty remains associated with the utility values used in our model. Nevertheless, data from the one-way sensitivity and scenario analyses demonstrated that even when extremely favourable utility values were assigned, the overall conclusion regarding the poor economic value of the intervention remained unchanged.

Last, our study focused on comparing the costs and outcomes between atezolizumab/nab-paclitaxel and nab-paclitaxel, the treatment arms used within the IMpassion130 trial. While beyond the scope of this study, further research is warranted to assess the cost-effectiveness of adding atezolizumab to other chemotherapies commonly used in practice, upon the emergence of relevant clinical data. For instance, compared with nab-paclitaxel, solvent-based paclitaxel is more frequently used owing to its lower cost. Of note, a phase III randomised trial is currently underway to elucidate the efficacy of adding atezolizumab to solvent-based paclitaxel for the treatment of locally advanced or metastatic TNBC (IMpassion131) and is estimated to complete in mid-2021 [31]. Furthermore, the availability of generic nab-paclitaxel could also improve the ICER of the combination regimen of atezolizumab/nab-paclitaxel given much lower cost of generic nab-paclitaxel.

## Conclusion

In conclusion, our study demonstrated that at the current cost of treatment, atezolizumab/nab-paclitaxel combination therapy did not represent good value for limited healthcare dollars compared to nab-paclitaxel monotherapy for patients with advanced, PDL1-positive TNBC, from the Singapore healthcare system perspective. Our findings will be important in informing policy makers in making funding decisions alongside other considerations such as comparative effectiveness, unmet need and budget impact.

## Data Availability

All data generated or analysed during this study are included in this published article.

## Abbreviations

AE: Adverse events
BSA: Body surface area
CT: Computed tomography
HER2: Human epidermal growth factor receptor-2
ICER: Incremental cost-effectiveness ratio
KM: Kaplan Meier
LY: Life year
nab: Nanoparticle albumin-bound
NSCLC: Non-small cell lung cancer
PDL1: Programmed death ligand 1
PD: Progressed disease
PF: Progression-free
PFLY: Progression-free life year
PFS: Progression-free survival
OS: Overall survival
QALY: Quality-adjusted life year
TNBC: Triple-negative breast cancer
